# Biological isolation and characterization of *Catharanthus roseus* (L.) G. Don methanolic leaves extracts and their assessment for antimicrobial, cytotoxic, and apoptotic activities

**DOI:** 10.1186/s12906-022-03810-y

**Published:** 2022-12-09

**Authors:** Somashekara Rajashekara, Dondapati Reena, Mullahalli Venkataramareddy Mainavi, Locheruvapalli Srinivasa Sandhya, Utpal Baro

**Affiliations:** Department of Studies in Zoology, Centre for Applied Genetics, Bangalore University, Jnana Bharathi Campus, Off Mysuru Road, Bengaluru, 560 056 India

**Keywords:** Antibacterial activity, Anticancer activity, Apoptosis, Methanolic leaf extracts, MDA-MB-231 cell lines

## Abstract

**Background:**

Biological synthesis of natural products from plants has made us an inspiring methodology in the field of science and biotechnology.

**Methods:**

The methanolic extracts of *Catharanthus roseus* (L.) G. Don plant leaves (CrPLE) were extracted and characterized by utilizing the phytochemicals estimation, Thin-layer chromatography (TLC), and High-Performance Liquid Chromatography (HPLC) analysis; and further evaluation for an understanding of the biomedical uses of CrPLE was done.

**Results:**

The evaluation of the seven phytochemicals designates the presence of secondary metabolites in the CrPLE. The CrPLE (test samples) exhibited the Catechin and Caffeic acid contents of 0.0055 and 0.0149 mg/g respectively. The CrPLE revealed the highest antimicrobial activity and showed a mortal effect against the tested microorganisms. Cytotoxicity of the breast cancer cell lines was exposed that CrPLE as a respectable anticancer specialist and metabolically vigorous cells.

**Conclusion:**

Consequently, the biological synthesized methanolic leaf extracts of the *C. roseus* plants would be appreciated and have incredible contributions to the field of medicinal applications.

**Graphical Abstract:**

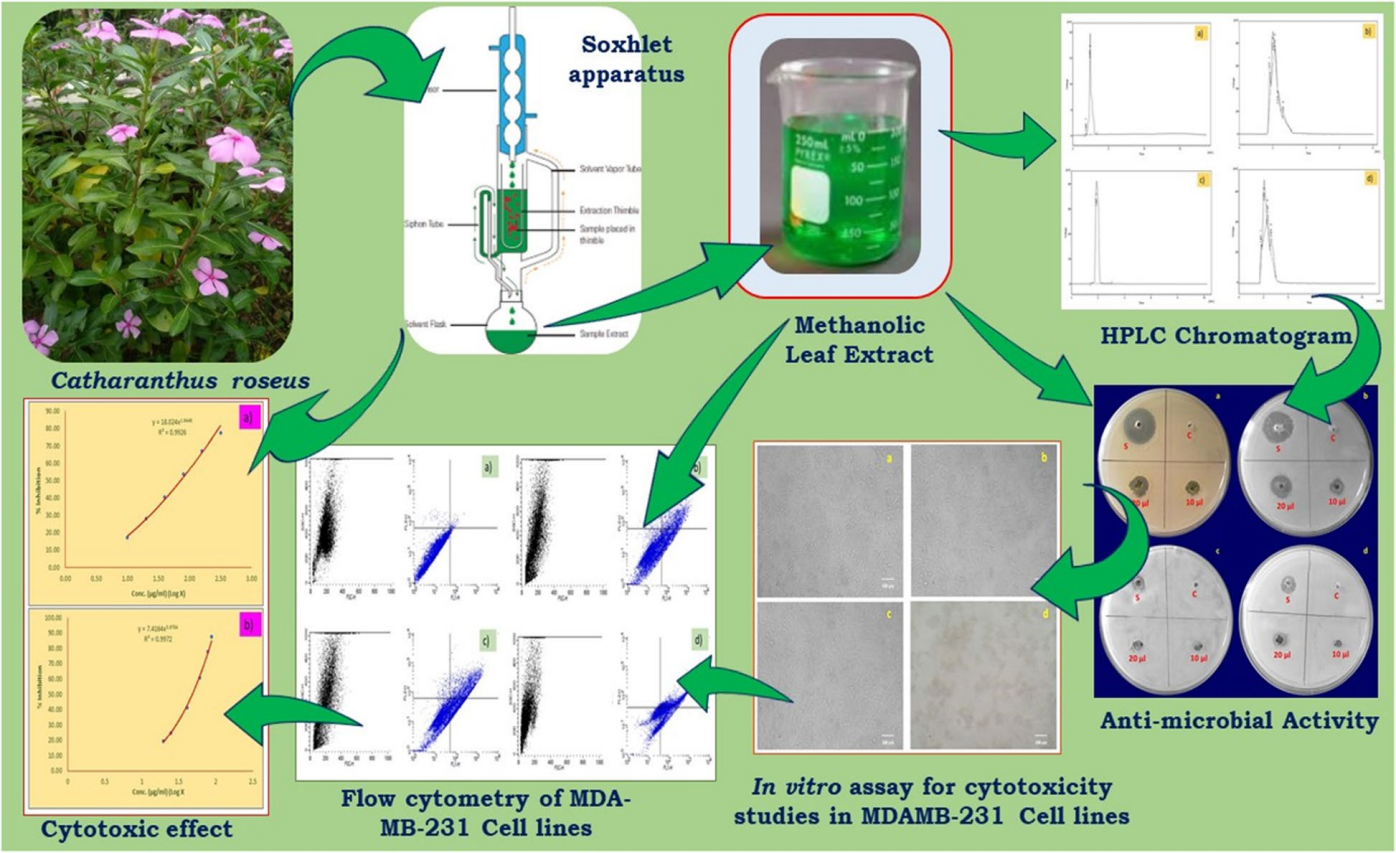

## Highlights

• The methanolic leaf extracts of the *Catharanthus roseus* plants contain secondary metabolites.

• CrPLE extracts showed antimicrobial and anticancer activities.

• The use of *Catharanthus roseus* plants as a “natural herbal resource” in the pharmaceutical and pharmacological industry has been validated and further expanded.

## Background

India has a wide and interesting variety of different biodiversity of beneficial plant sources that are still yet to be investigated totally. Because of the cost and higher optional impacts connected with the misleadingly manufactured prescriptions, the necessity for novel medication stuffs out by the plant achieved an uncommon notice in the current investigation [15].

*Catharanthus roseus* (L.) G. Don (Gentianales: Apocynaceae), regularly recognized as *Vinca rosea*, Rosy or Pink Periwinkle, and Madagascar Periwinkle, a persistent species that are started in Madagascar and are local to Madagascar and Southern Asia [12]. This plant is one of the generally utilized restorative plant and decorative plants [35, 44]. These plants are disseminated all through the biosphere creating under a wide extent of climatic circumstances and they are known to overwhelm or to convey accommodating feed and food things. Research scholars identified the occurrence of alkaloids in *C. roseus*, this plant was thoroughly examined with drug associations [12]. The seeds and leaves of *C. roseus* are applied as normal medications and antimicrobial activities [52]. Since the roots of these plants are the primary wellsprings of the counter hypertension alkaloid ajmalicine [5], the concentrates are utilized in the treatment of hypertension, improvement in the cerebral course, and obstructive circulatory sicknesses [62].

*Catharanthus roseus* displays as an anticancer or anti-carcinogenic agent [13, 30, 38, 50, 58, 60] and antioxidant agents [19, 20]. Too the ornamented regard, the *C. roseus* plant is perceived to have different pharmacological exercises of medical importance, for example, mitigating, cancer prevention agent, and anticarcinogenic activities [6, 60]; antibacterial action [17, 59]; antimitotic activity [1]; antitumor activity [64, 65]; antidiabetic activity [16, 67]; antifeedant and larvicidal activities against *Earias vittella*, larvicidal on *Culex quinquefasciatus* (house mosquito) and *Aedes aegypti* (yellow fever mosquito) [46, 47]. Various pieces of plant structure an expected spice to fix different diseases and issues, for example, therapy of a few sicknesses like diabetes, jungle fever, and Hodgkin’s lymphoma [1, 23], to regulate drain and scurvy, as a gargle for toothache, and recuperating and cleaning of persistent injuries, to treat diabetic ulcer and oral hypoglycemic specialist [2]. For the therapy of numerous infections, utilize this restorative plant like injuring pressure [14, 61]; therapy of hypertension and circulatory problems [7, 8]; mending purposes [31]; prophylactic specialist in a significant number of the plague sicknesses [45]; therapy of malignant growth and diabetics [29].

*Catharanthus roseus* has numerous pharmacological impacts. Hence, the plant (roots, shoots, and leaves) extracts the plant parts extricates are being utilized against a few infections like loose bowels, Alzheimer’s illness, asthma, hacks, throat infections, sore throat, the anticipation of dementia, water maintenance (edema), diarrhea, ailment, fart, tuberculosis, dyspepsia, tonsillitis, chest torment, digestive torment, toothache, bug’s sting, for outside practice to treat skin issues such as dermatitis, skin inflammation as well as skin break out, expanding (mitigating), mind stimulatory activities, cardiotonic, CNS depressant, hostile to angiogenesis impacts, hostile to feeding, against sterility, against malarial, likewise an intense enemy of microbial, hostile to oxidant action, have against malignant growth, hostile to diabetic, cytotoxic, hypertension, hypolipidemic action and so on [56]. This plant had a significant wellspring of restorative mixtures fundamentally worried to the fields of ethnobotany, pharmacology, phytochemistry, and natural action chemotherapeutic medications and treatment.

Several engineered mixtures of *C. roseus* are utilized as prescriptions and in this manner, plants are filled in as an elective source nowadays [23]. In any case, not very many reports are accessible against the breast disease cells and the greater part of these investigations are essentially founded on the natural amalgamation. At present, there has just been a restricted information presence for the cytotoxic impacts of organically combined separates towards particular human breast cancer cells. In the persistence of the previous work, we have made an investigation of the *C. roseus* plant for its capacity to biosynthesize the methanolic leaf extractions. A major aim of this study is to assess the cytotoxic impact of biosynthesized methanolic leaf extracts against human breast cancer cell lines. Our gathering has endeavored to investigate the biogenic synthesis of methanolic leaf extracts from *C. roseus* plants for biomedical practices.

With such an objective, an undertaking has been thru to join the methanolic extracts exploiting usually existing ordinary things by aapplying the *C. roseus* plant leaves. The leaves of *C. roseus* show supportive and automatic activities because of the occurrence of pharmacological and phytochemical activities [15, 43]. Consequently, current examination shows antimicrobial and apoptotic exercises of the isolated *C. roseus* plant leaves extractions (CrPLE) against the bacterial strains - *Escherichia coli* - ATCC 8739*, and Staphylococcus aureus* - ATCC 6538*; Aspergillus niger* - ATCC 6275 and *Candida albicans* - ATCC 10231. Further, the cytotoxic impacts of a methanolic method of the *C. roseus* plant leaf extractions (CrPLE) were tried against MDA-MB-231 cell lines. The current examination opens an innovative opening to utilize this strategy for an eco-approachable for the planning and biomedical applications of CrPLE.

## Methods

### Selection and collection of plant materials

**Fig. 1:**
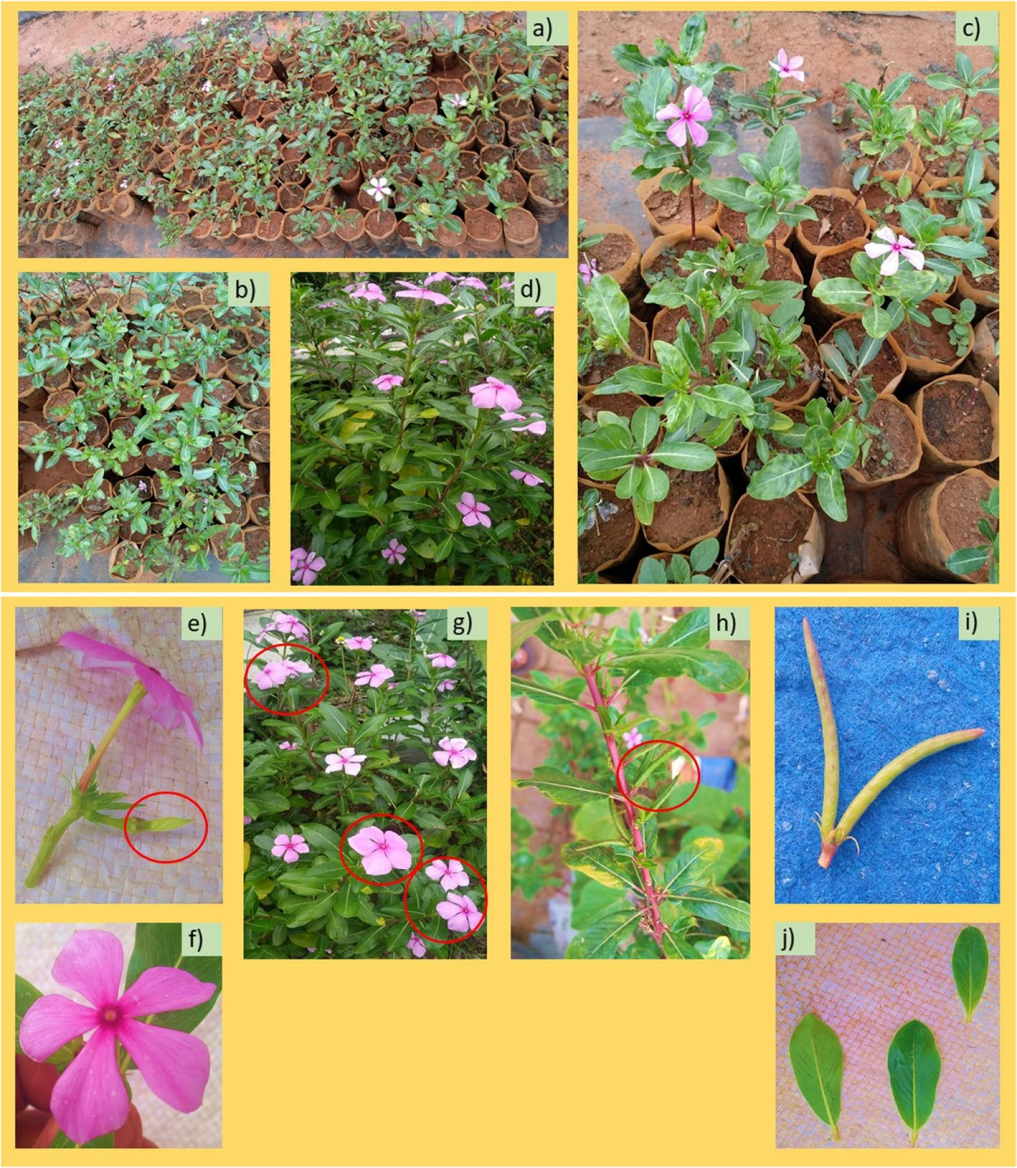
Photographs showing the site of morphological identifications and sample collections of the *Catharanthus roseus* plants (images that are depicted in Fig. 1 are taken by Rajashekara et al). **a**-**c** nursery site of plant rearing; **d** Leaves collected from the plants for the present experimentation; **e** Flower bud; **f** A close look up of Flower; **g** Wild types with purple flowers; **h** Fruits in the composed form with **i** two free narrow cylindrical follicles; **j** A closed view of leaves

*Catharanthus roseus* is a flowering plant species, local and endemic to Madagascar, yet developed somewhere else as a fancy and restorative plant. The plant materials were cultivated and followed by using the standard guidelines of plant nurseries of Karnataka State Forest Department, Karnataka State. One such nurseries is well-known as “Dhanavantri Vana”, and generally recognized as ‘Medicinal Plants of Paradise’. Fresh and healthy cultivated leaves of *C. roseus* plants (Fig. 1) were gathered from the nurseries of the ‘Medicinal Plants of Paradise’, Jnana Jyothi Nagar, Jnana Bharathi, Bengaluru (12° 56′ 30.67“ N and 77° 29’ 53.85” E with an elevation of 800.41 m asl), Karnataka 560,056, India. Congregated leaves were presented to the affirmation process and identified by Dr. H. R. Raveesha, Professor in the Department of Botany, Bangalore University, Bengaluru 560,056, Karnataka, India. Also, a voucher specimen of this material was deposited and now, it is made available in an herbarium of the same Botany Department, Bangalore University. A vouchering reference number CRDVSR00VII0B021 has been provided by the Botany Department.

### Preparation of plant leaf extract using methanolic technique

Methanolic technique for the *C. roseus* plant leaves extractions (CrPLE) were experimented with as described by Kumar et al. [28], Koel et al. [26], and Syeda and Riazunnisa [59]. Weighed 20 g of desiccated sample powder and disintegrated in 100 ml of methanol in a 500 ml container with covering by aluminum foil. Then, at that point, the container was placed in a hot steam bath at 50 ° C for 4 h. After keeping aside, the extract was separated using Whatman strain removing paper and the remainder was gathered in a 50 ml container. Filtrate present over the strain-removing paper was disposed of and the remainder was engaged for additional utilization. Then the remainder was kept at 50 °C for a few hours until the extract got completely dehydrated and turned into semi-solid form. This semi solid sample was weighed and the yield was observed. The methanolic leaf extractions of *C. roseus* were determined utilizing the formula:

$$\%\;\text{yield}=\left(\text{Dry}\;\text{extract}\;\text{weight}\right)/\left(\text{Total}\;\text{sample}\;\text{weight}\right)\;\text{X}\;100$$

### Characterization of the CrPLE

#### Determination of phytochemicals screening

The phytochemical screening of achieved methanolic leaf extracts of the *C. roseus* plants was experimented with as endorsed by van Der Heijden et al. [60], Nayak and Pereira [39], Jaleel et al. [18], and Govindasamy and Srinivasan [17]. The screening was performed for the sign (whether present or absent) of phytochemicals through the diagnostic tests by the color concentration or the precipitate formation. The results were qualitatively determined and stated as a positive ‘+’ sign for the appearance and a negative ‘−’ sign for the nonappearance of phytochemicals [17, 49].

#### Thin-layer Chromatography (TLC) analysis of the methanolic leaf extracts of C. roseus plants

Thin-layer chromatography (TLC) was accomplished the survey to the known purity of *C. roseus* plant leaves extracts (CrPLE) [28, 63]. 10 mg/ml samples were ready for experimentation. 2.5 μl of the sample was marked on a TLC plate and permitted to dry. A TLC plate is comprised of a slim layer of Silica gel (0.25 mm) with fluorescent indicator F_254_ with the dissolvable system. Chloroform: methanol (9.5: 0.5) was employed as a solvent system for TLC analysis. The strip or plate is then positioned with this end dunking into the dissolvable blend, taking into consideration that the example spot/zone isn’t drenched in the dissolvable. As the dissolvable moves towards the opposite finish of the strip, the test combination isolates it into different parts. This is called as improvement of TLC plates. The division relies upon a few elements. The plate is taken out after an ideal advancement time and dried, and the spots/zones are identified utilizing a UV chamber and the Rf value is determined utilizing the accompanying formulae:

$$\textrm{Retention}\ \textrm{factor}\ \left(\textrm{Rf}\right)\kern0.5em \textrm{value}=\frac{\textrm{Distance}\ \textrm{moved}\ \textrm{by}\ \textrm{the}\ \textrm{compound}}{\textrm{Distance}\ \textrm{moved}\ \textrm{by}\ \textrm{the}\ \textrm{solvent}}$$

In a silica sheet, the mark is made with a pencil about 1 cm range from the lower edge of the end. The fluid concentrate sample of *C. roseus* leaves was marked online, with the assistance of a fine capillary. The distance moved by the solvent is set apart with a pencil and the spots are seen by a UV lamp and set apart as the shape and color they make. The distance traveled by the spots and the distance traveled by the solvent are noted and the retention factor (Rf) value of each spot is determined [63]. The solvent ascent was fixed at 10 cm in all cases [28]. The Rf values of all the TLC plates were then determined.

#### High-Performance Liquid Chromatography (HPLC) analysis of the methanolic leaf extracts of C. roseus plants

Raw extract (10 mg/ mL), methanol chloroform, and hexane (based on solubility) from a stock of *C. roseus* leaves were liquified to the concentration of 1.5 mg/mL, then sieved through a 0.45 μm nylon Phenex syringe strain remover and investigated utilizing a Shimadzu HPLC system (Shimadzu, Kyoto, Japan) fixed with a reversed-phase column C18 column (Synergi 4u Polar-RP80A Column 250 x 4.6 mm 5um) (Torrance, USA) utilizing UV detection (UV-VIS detector SPD-10A) set at 210 nm and 280 nm. The portable phase consisted of 3% (v/v) acetonitrile in 0.2% (v/v) orthophosphoric acid (Solvent A) and absolute acetonitrile (Solvent B). An auto-injector (SIL-10A HT) was utilized to inject 20 μL sample volumes onto the HPLC at a flow rate of 1 mL/min. with a incline elution plan as follows: 0–10 min., 0% B; 10–30 min., 50% B; 30–45 min., 50% B; 45–55 min., 60% B; 55–60 min., 0% B [49]. Chemical mixtures that existed in various extracts of *C. roseus* were resolved trailed by utilizing a technique of Bramati et al. [4], Pan et al. [42], Wagay et al. [63], Lin et al. [32], Liu et al. [34] and Deguchi et al. [9].

Reference guidelines including caffeic acid and catechin were manufactured in methanol at the concentration of 200 μg/mL for recognition and measurement of the specific substances confined in the extracts. Peaks were recognized in light of the retention time of caffeic acid and catechin standards. Complete coloring matters as a percentage of content was determined from the total peak area by adding from the adjustment diagram [33]. Unidentified individual mixtures were evaluated involving Caffeic acid and Catechin as a source of the perspective norm at 250 and 278 nm respectively and the result were expressed as μg equivalents per ml of extract (μg/ml) [33, 49]. The diagnostic signs were observed at 2–20 mV latent functional. The following equation was utilized to regulate the concentrations of the substances present in the extracts

$$\textrm{X}\frac{mg}{L}=\left\{\textrm{Area}\ \textrm{of}\ \textrm{sample}\div \textrm{Area}\ \textrm{of}\ \textrm{standard}\right\}20\ \textrm{mg}/\textrm{L}$$

Along these lines, a purified solution was examined by a high-performance liquid chromatography system.

#### Cell line culture and preparation

Human breast cancer MDA-MB-231 strain -CRM-HTB-26 cell line was acquired from the American Type Culture Collection (ATCC) stock cells, then this was cultivated in Dulbecco’s modified eagle’s medium (DMEM) enhanced with 10% deactivated Fetal Bovine Serum (FBS), penicillin (100 IU/ ml), streptomycin (100 μg/ ml) in a moistened atmosphere of 5% CO_2_ at 37 °C pending blended. The cell lines were separated with the cell separating solution (0.2% trypsin, 0.02% EDTA, and 0.05% glucose in PBS). The feasibility of the cells was monitored and centrifuged. Additionally, the 50,000 cells/ well were cultivated in a 96 well plate and nurtured for 24 h at 37 °C, a 5% CO_2_ incubator.

### Medicinal practices of the biologically synthesized methanolic leaf extracts of *C. roseus* plants

Assessment for anti-microbial action, cell cycle studies by flow cytometry, *in vitro* assay for cytotoxicity, and anticancer action by apoptosis tests utilizing prepared methanolic leaf extracts of *C. roseus* plants were experimented by succeeding the beneath referenced events.

#### Anti-microbial activity

Minimum inhibitory concentration (MICs) is the minimum dosage of a compound prevent microorganism development and was calculated based on cultures comprising a changeable dosage of the methanolic leaf extracts of *C. roseus* plants (CrPLE) utilizing the agar well diffusion technique [11, 49, 59].

The anti-microbial present in the plant extract is permitted to diffuse out into the medium and communicate in a plate newly cultivated with the test organisms. The resulting zones of inhibition would be consistently roundabout and the distance across of zone of inhibition can be measured in millimeters. The agar utilized was Meuller-Hinton agar for bacteria, Potato Dextrose agar for fungi, and Yeast Peptone Glucose (YEDP) Agar for yeast that is thoroughly tried for configuration and pH. This strategy is fine reported as the standard sectors of inhibition have been calculated powerless in addition to safe qualities.

Petri plates (with a diameter of 90 mm) comprising 20 mL Meuller-Hinton agar were cultivated utilizing cotton swabs with 24 h (old) culture of the microbial strains. Wells were cut (10 mm diameter) and 50 μL of various concentrations of a test sample. The plates were then nurtured at 37 °C for 24 h. The anti-microbial action was examined by estimating the diameter of the inhibition zone shaped around the well against the test microorganisms [51, 53]. Moreover, *Staphylococcus aureus* - ATCC 6538 and *Escherichia coli* - ATCC 8739 cell suspension were ready and grown on tryptone soup, and cultures were nurtured for 24 h at 37 °C. The cell suspensions of the cultures were acclimated to 1–2 x 10^6^ cells/ ml. The methanolic leaf extracts of *C. roseus* plants are utilized as the test compounds with samples (20 μl and 10 μl), standard as Ciprofloxacin (20 μl) for *S. aureus* and *E. coli* were added to the 5 mm well on agar plates [57]. Besides, *Aspergillus niger* ATCC 6275 and *Candida albicans* ATCC 10231 spore suspension were ready and developed on potato dextrose broth, and cultures were nurtured for 5 to 7 days at room temperature (27 °C). Once more, the methanolic leaf extracts of *C. roseus* plants are utilized as the test compounds with samples (20 μl and 10 μl), standard as Itraconazole (20 μl) for *A. niger* and *C. albicans* were added to the 5 mm well on agar plates.

After incubation, the experimented plates were detected for a zone of inhibition around the wells. The antimicrobial action was assessed by estimating the diameter of the inhibition zone of the tested microorganisms [3, 45, 51, 63].

#### Cytotoxicity studies

Levels of cells in different phases of the cell cycle with the mixtures of treated and untreated (controlled) populations were estimated through flow cytometry utilizing the FACS Caliber (BD Biosciences, San Jose, CA).

An *in vitro* assay typically experimented with the monolayer cell culture was trypsinized and the total of cells was acclimated to 5.0 x 10^5^ cells/ ml utilizing separate media encompassing 10% FBS in half-area flat-bottomed microtiter plates. To each well of the 96-well microtiter plate, 100 μl of the watery cell suspension (50,000 cells/ well) was supplemented. Later 24 h, when a partial monolayer was shaped, the supernatant was spun off, splashed the monolayer once with medium and 100 μl of various trial dosages of trial samples were supplementary to the fractional monolayer in microtiter plates.

The plates were then nurtured at 37 °C for 24 h in a 5% CO_2_ atmosphere. The decrease of tetrazolium salts was broadly acknowledged as a solid method for inspecting cell expansion. The yellow tetrazolium MTT (3-(4, 5-dimethyl thiazolyl-2)-2, 5-diphenyltetrazolium bromide) was diminished through metabolically dynamic cells, partially employing the activity of dehydrogenase catalysts, to create decreasing counterparts, for instance, NADH and NADPH. The resultant intracellular purple formazan can be solubilized and evaluated thru spectrophotometric analysis. Test estimates the cell multiplication amount and then again, when metabolic occasions lead to programmed cell death or corruption, the decrease in cell viability.

Cells cultivated in T-25 flasks were trypsinized and suctioned into a 5 mL centrifuge tube. The cell pellet was acquired by centrifugation at 300 x g. The total of the cell was changed, utilizing DMEM HG medium, with the end goal that 200 μl of suspension confined around 10,000 cells. To each well of the 96 well microtitre plate, 200 μl of the cell suspension was supplemented and the plate was nurtured at 37 °C and 5% CO_2_ atmosphere for 24 h.

Later 24 h, the spent medium was aspirated. 200 μl of various test dosages of test drugs were supplemented to the respective wells. The plate was then incubated at 37 °C and 5% CO_2_ atmosphere for 24 h. The plate was eliminated from the incubator and the drug-containing media was suctioned. 200 μl of medium encompassing 10% MTT reagent was then supplemented to each well to get a final concentration of 0.5 mg/mL and the plate was nurtured at 37 °C and 5% CO_2_ atmosphere for 3 h. The culture medium was eliminated deprived of upsetting the crystals designed. Then 100 μl of solubilization solution (DMSO) was supplementary and the plate was caringly shaken in a gyratory shaker to solubilize the formed blue formazan [37].

The optical density was estimated utilizing a microplate reader at a wavelength of 570 nm and 630 nm. The percentage growth inhibition was determined, after deducting the background and the absolute, and the dosage of the test drug expected to prevent cell growth by 50% (IC_50_) values was produced from the concentration-response curve for the cell line [10, 53].

The percentage of viability was calculated utilizing the formula:

$$\%\;\text{Cell}\;\text{viability}=\left[\left(\text{At}-\text{Ab}\right)/\left(\text{Ac}-\text{Ab}\right)\right]\;\text{X}\;100$$

where A_t_ = absorbance value of a trial substance, 

A_b_ = absorbance value of absolute and 

A_c_ = absorbance value of control.

#### Mechanism of cell death: apoptotic assay

Apoptosis is a cell death interaction considered through morphological and biochemical structures happening at numerous phases. As soon as set off, programmed cell death continues through various energy relying upon cell types and comes full circle with cell disturbance and arrangement. A basic phase of programmed cell death includes securing exterior fluctuations by kicking the bucket cells that at last outcomes in the acknowledgment as well as the take-up of these cells by phagocytes.

For programmed cell death induction, the MDA-MB-231 cell lines were splashed several times in DMEM cell culture medium and then cultivated in 6-well plates at 5 X 10^5^ cells/ mL in DMEM cell culture with 1% FCS as defined by Koopman et al. [27]. To recognize cells that had lost membrane integrity, Propidium Iodide (PI) dye was supplementary to the last concentration of 10 txg/ ml before the examination [36].

The day preceding enlistment of apoptosis, plated 1 X 10^6^ cells for every well for a 6-well plate utilizing DMEM cell culture medium. Later ~18 hours, the wells for drifting (dead) cells and dispensed with by pipette. Supplanted with a new culture medium to the original volume. Offered cells actuate apoptosis with 40 and 80 μg/ml of *C. roseus* leaves, and incubate for 24 h. Afterward, gathered cell culture medium into 15-mL tubes. Utilizing a policeman, the cells were confined from the dish and added 1 mL of medium to each well, and transported the contents to the 15-mL tubes. Centrifuged and disposed of the supernatant. Splashed the cells twice with cold PBS and afterward resuspend cells in 1 mL 1X Binding Buffer at a dosage of ~1 x 10^6^ cells/mL. 500 μL of cell suspension is aliquoted and 10 μL of PI and 5 μL Annexin V is supplemented. The suspension is incubated for 15 minutes at RT in the dark. Post incubation, the cells were examined through a flow cytometer at the earliest opportunity (within 1 hour).

## Results

### Biological synthesis of the methanolic leaf extracts of the *C. roseus* plants (CrPLE)

The biological synthesis of natural products has made us a thrilling methodology in the arena of the usual science of natural science and biotechnology. The methanolic leaf extracts of the *C. roseus* were prepared and finally yielded 2.89 g from the 20 g of the dried sample powder.

### Characterization of the CrPLE

#### Determination of phytochemicals screening

The phytochemicals existing in the methanolic extracts of the *C. roseus* plant leaves (CrPLE) perform as redox agents and the results were qualitatively determined as ‘positive’ signs for the presence and ‘negative’ signs for the absence of phytochemicals. Among the 12 tested phytochemicals screening, the existence of seven phytochemicals for instance alkaloids, carbohydrates, flavonoids, glycosides, phenols, steroids, and tannins indicate the clear existence of secondary metabolites in the CrPLE (Table 1).

**Table 1:** Phytochemical analysis for the methanolic leaf extracts of the *Catharanthus roseus*

No.	Phytochemicals	Methanolic extract of leaves
1.	**Alkaloids**	+
2.	**Carbohydrates**	+
3.	**Tannins**	+
4.	**Terpenoids**	–
5.	**Glycosides**	+
6.	**Steroids**	+
7.	**Saponins**	–
8.	**Flavonoids**	+
9.	**Glycoproteins Test**	**–**
10.	**Volatile oils**	–
11.	**Starch**	–
12.	**Phenol**	+

+ and - indicate presence and absence of the phytochemical groups

#### Thin Layer Chromatography (TLC) analysis

**Fig. 2:**
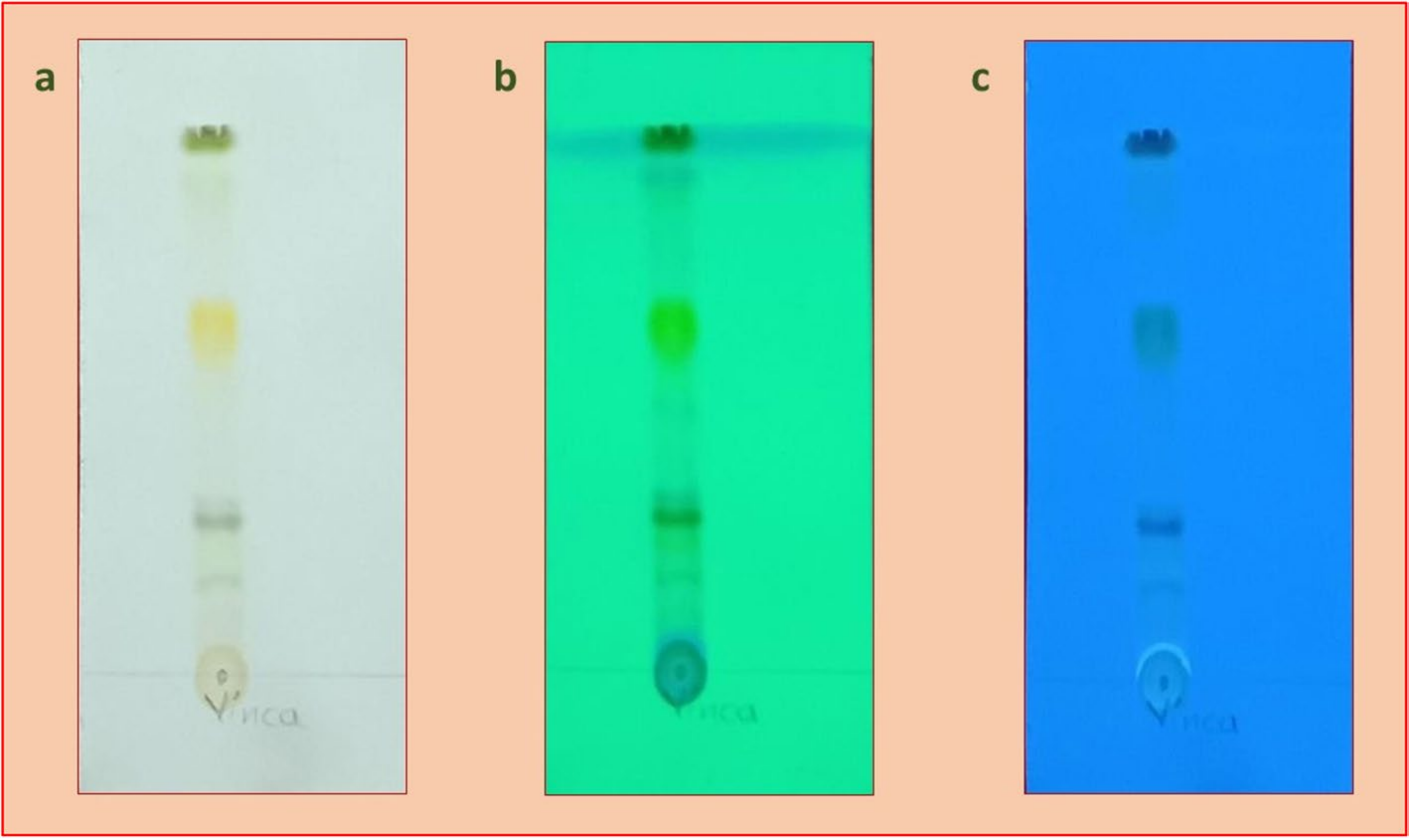
TLC chromatogram of the crude *Catharanthus roseus* leaf extracts at different nm wave crude **a** visible light **b** 254 nm and **c** 366 nm

Thin Layer Chromatography analysis of methanol extracts of *C. roseus* leaves displayed the occurrence of dynamic compounds that were pictured in the various frequency of wavelengths (UV and Visible lights). The methanol and chloroform (solvent systems) are polar and non-polar solvents respectively and are used for the identification of straining in the various amalgamations constructed on the polarity of the substance and their ratio, the number of bands obtained, and their retention factor (Rf) value is given in the following Table 2. From the results, it was observed that the leaf solvent system extract revealed more than two bands. Blue and light blue color pigmentation were detected in the leaf extracts (Fig. 2) might be because of the presence of the pigments. Crude leaf extracts run quickly in the TLC plate when related to the water. The leaf extracts were light in color consequently it is challenging to decipher the example run when put inside the dissolvable framework. Leaf RF acquired means the extremity of the isolated groups, bigger the Rf values bring down the extremity of the mixtures as well as the other way around. That is, the Rf esteem got is conversely corresponding to the extremity of the compound.

**Table 2:** TLC characteristics for the *Catharanthus roseus* crude extraction

Sample	Solvent	TLC Band @ 254 nm	Retention Factor (Rf)	TLC Profile characteristics		
				Visible Light	Visible Light 254 nm	Visible Light 366 nm
*Catharanthus roseus* leaf extract	**Methanol**	**11**	0.77	Brown	Light green	–
			1.09	Yellow	–	Light blue
			1.30	Brown	Light green	Light blue
			1.51	Brown	Green	–
			1.69	Yellow	Blue	Light blue
			2.01	Brown	Blue	Light blue
			2.46	Brown	Light green	Light blue
			3.07	Yellow	–	Light blue
			3.63	Brown	Light green	–
			4.18	–	Light green	Light blue
			4.47	Green	–	Light blue
			1.00	–	Light green	Light blue

#### High-Performance Liquid Chromatography (HPLC) analysis of the CrPLE

**Fig. 3:**
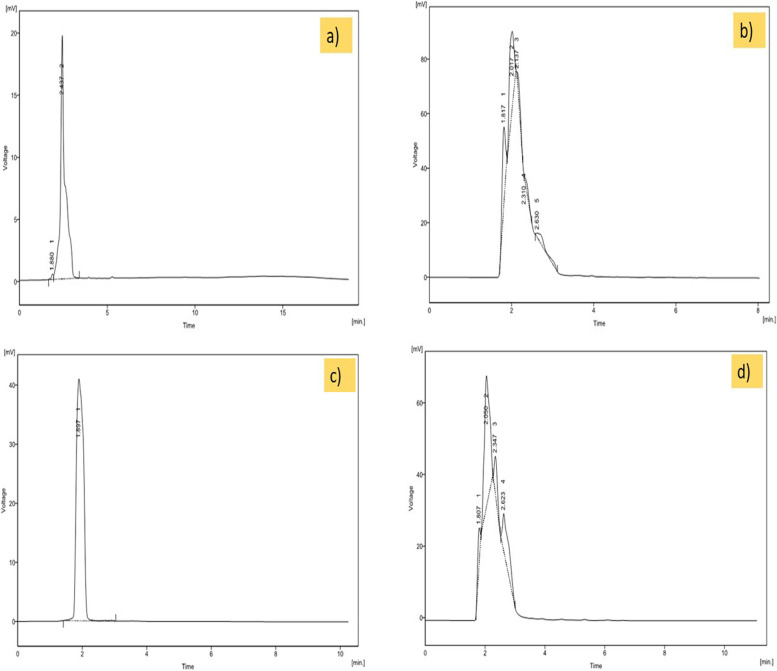
High Performance Liquid Chromatography (HPLC) Chromatogram of **a** Standard Catechin (100 ug/ml) identified in; **b** methanolic leaf extracts of the *C. roseus* plants (test sample) analyzed at a wavelength of 250 nm; **c** Standard Caffeic Acid (100ug/ml) identified in; and **d** methanolic leaf extracts of the *C. roseus* plants (test sample) analyzed at a wavelength of 278 nm

The absorption maxima in UV-spectrophotometric analysis were recorded at 250 nm which helped in easy analysis *via* HPLC analysis. Analysis by linear phase chromatography using a methanol-water gradient system revealed the presence of a solo top, with a retaining time of standard (catechin) and sample (methanolic leaf extracts of the *C. roseus* plants) is 2.437 and 2.310 min respectively (Fig. 3a and b, and Table 3). In addition to this, analysis by linear phase chromatography using a methanol-water gradient system revealed the presence of a solo top, with a retaining time of standard (caffeic acid) and sample (methanolic leaf extracts of the *C. roseus* plants) is 1.897 and 1.807 min respectively (Fig. 3c and d, and Table 3). The catechin content and caffeic acid content in test samples are summarized in Table 3. Finally, the methanolic leaf extracts of the *C. roseus* plants (test sample) displayed catechin and caffeic acid contents of 0.0055 and 0.0149 mg/g respectively. Both contents are the form of organic compounds present as a secondary metabolite of the *C. roseus* plants that consists of a phenolic functional group.

**Table 3:** HPLC characteristics for the Catechin and Caffeic Acid content for the methanolic leaf extracts of the *Catharanthus roseus* plants (test samples)

Samples	Stock	Retention time (min)	Area (mv*S)	Catechin Content ug/ ml of extract	Dilution factor	Catechin Content mg/ g of extract
**Catechin (ug/ ml)**	100	2.437	365.026	–	–	–
**Sample (*C. roseus*) (mg/ ml)**	10	2.310	20.130	5.51	1	0.0055
**Caffeic acid (ug/ ml)**	100	1.897	629.931	–	–	–
**Sample (*C. roseus*) (mg/ ml)**	10	1.807	54.240	14.86	1	0.0149

### Medicinal practices of the biologically synthesized methanolic leaf extracts of *C. roseus* plants

#### Anti-microbial activity

**Fig. 4:**
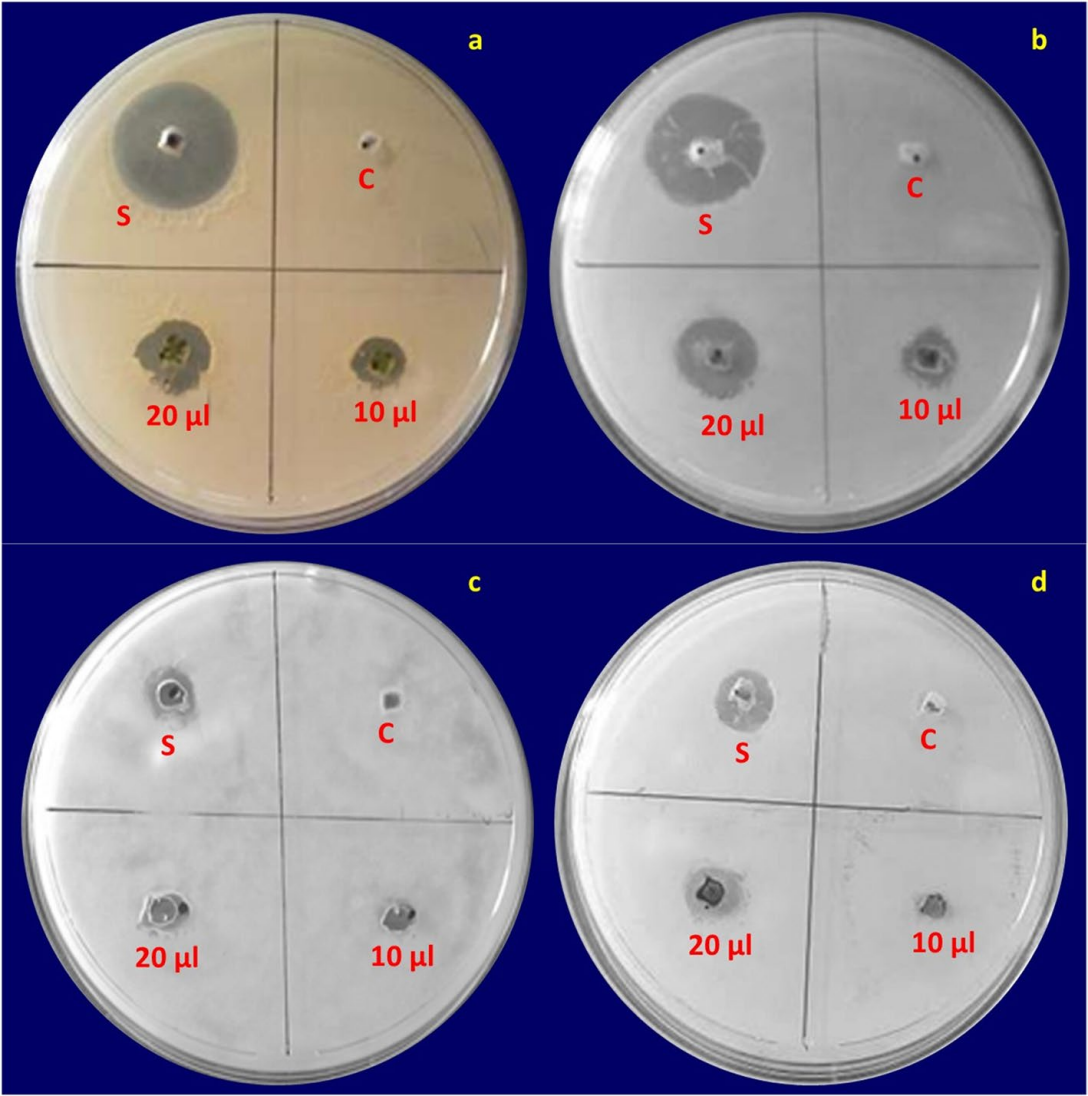
Antimicrobial activity of the methanolic leaf extracts of *Catharanthus roseus* using a zone of inhibition assay. (i) Antibacterial activity on **a** *Staphylococcus aureus* and **b** *Escherichia coli* with S: Standard Ciprofloxacin and C: Control (5% Methanol). (ii) Antifungal activity on **c** *Aspergillus niger* and **d** *Candida albicans* with S: Standard Itraconazole and C: Control (5% Acetone)

The methanolic leaf extractions of *C. roseus* have shown moderate to highest inhibitory activity against these mentioned microorganisms through the agar well diffusion technique (Fig. 4). A zone of inhibition was detected compared to the methanolic extracts of *C. roseus* leaves (as trial substances), Ciprofloxacin (as standard for antibacterial organisms - *Escherichia coli* and *Staphylococcus aureus* (Fig. 4a and b respectively)) and Itraconazole (as standard for antifungal organisms - *Aspergillus niger* and *Candida albicans* (Fig. 4c and d respectively)) are briefed in Table 4. The test sample exposed the highest antibacterial action against *S. aureus* (14 ± 0.0 mm) compared to the remaining studied species. Thus, the methanolic leaf extract of *C. roseus* plants exhibited the highest antimicrobial action and established with fatal outcome against the *S. aureus* at different dosages. Also, our study exposed the CrPLE exhibited substantial antimicrobial activities and therefore, could be useful for biological practices.

**Table 4:** Minimum Inhibitory Concentration (MIC) activity of the methanolic leaf extracts of *Catharanthus roseus* against the microbial species such as *Escherichia coli*, *Staphylococcus aureus*, *Aspergillus niger* and *Candida albicans*

Sample labels	Test organisms	Test compounds	Concentration (μg) per well	Zone of inhibition (mm)
***Catharanthus roseus*** **leaves**	*Staphylococcus aureus*	Control	5%	–
		Ciprofloxacin	2 μg	22 ± 0.0
		Sample	2 μg	14 ± 0.0
			1 μg	10 ± 0.0
	*Escherichia coli*	Control	5%	–
		Ciprofloxacin	2 μg	22 ± 0.0
		Sample	2 μg	12 ± 0.0
			1 μg	10 ± 0.0
	*Aspergillus niger*	Control	5%	–
		Itraconazole	20 μg	12 ± 0.0
		Sample	2 μg	09 ± 0.0
			1 μg	06 ± 0.0
	*Candida albicans*	Control	5%	–
		Itraconazole	20 μg	12 ± 0.0
		Sample	2 μg	10 ± 0.0
			1 μg	07 ± 0.0

#### Cytotoxicity studies

**Fig. 5:**
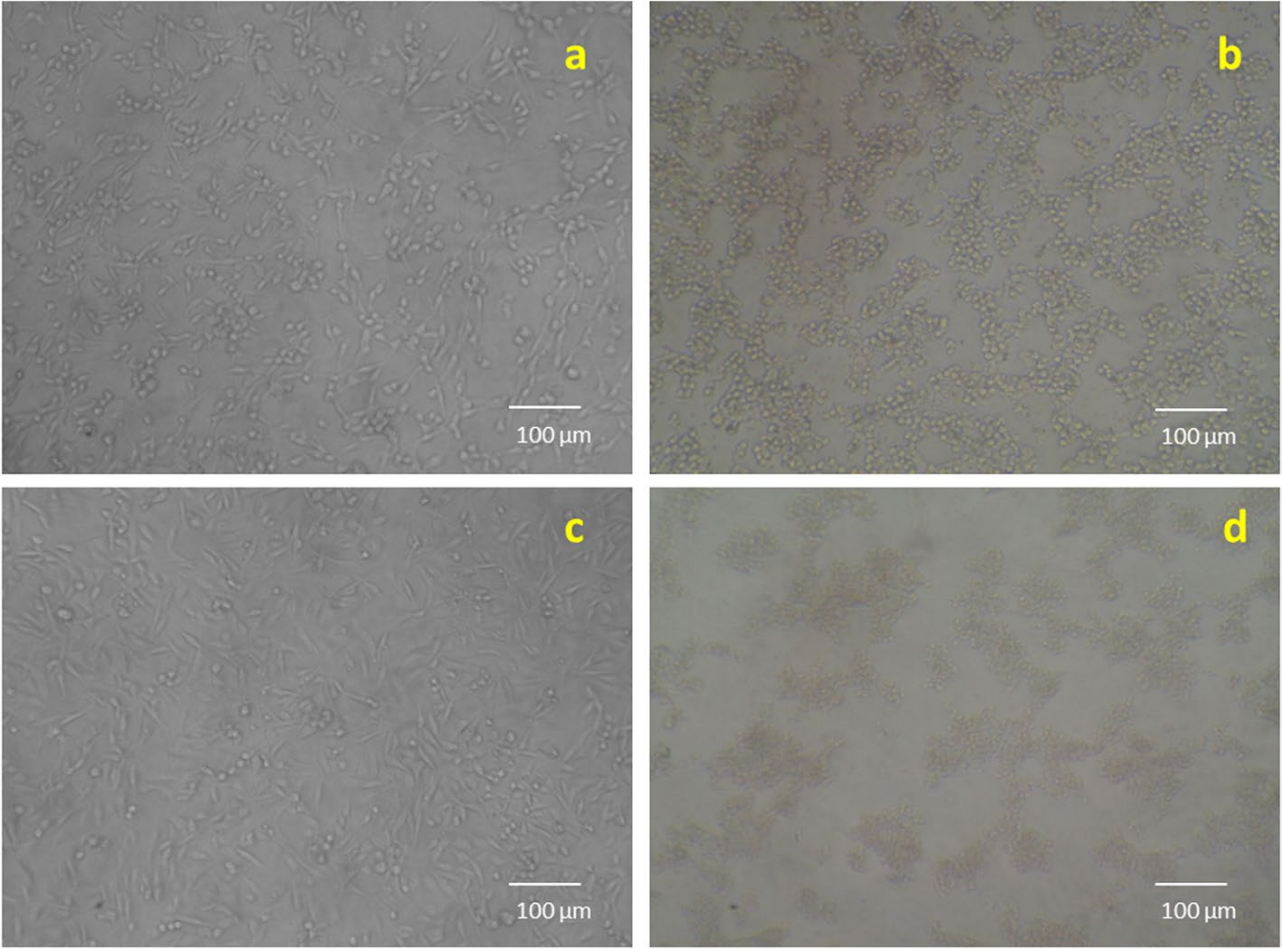
Morphology of MDA-MB-231 Cell lines after the exposure to methanolic leaf extracts of the *Catharanthus roseus* treated with the samples of **a** untreated sample, **b** standard as vinblastine, **c** treated with the samples of 10 μg/ml and **d** 320 μg/ml

**Fig. 6:**
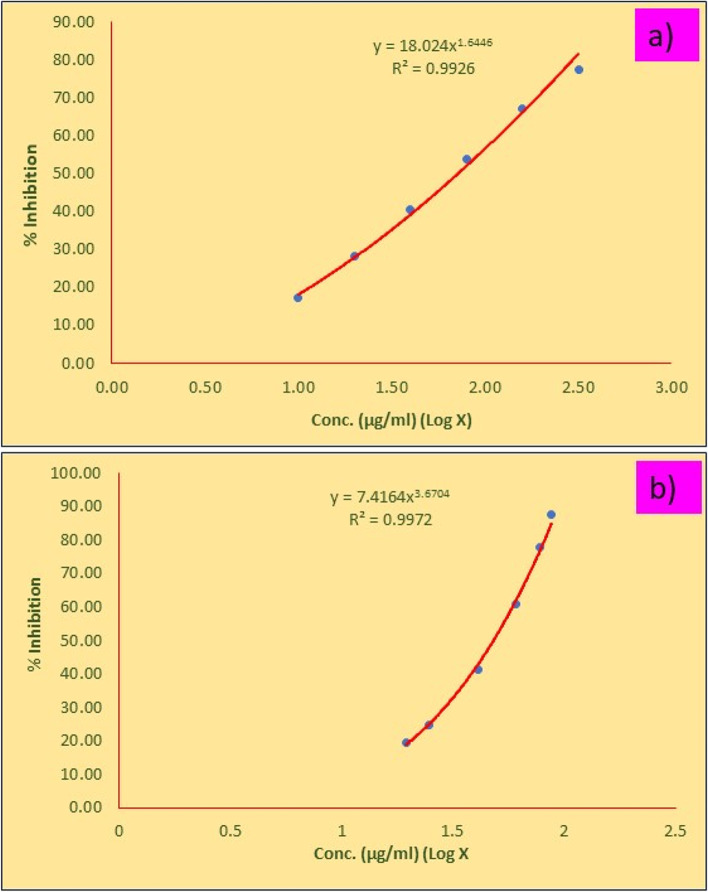
Cytotoxic effect of the methanolic leaf extracts of *Catharanthus roseus* against MDA-MB-231 cells lines; **a** Vinblastine **b** *C. roseus* plant leaves

Methanolic extracts produced from the *C. roseus* plant leaves exhibited cytotoxicity towards MDA-MB-231 cell lines (Fig. 5). Tested CrPLE showed the highest inhibition of cell propagation at 57.64 μg/ml (Table 5). The samples of methanolic leaf extracts of the *C. roseus* for 24 h treatment showed an IC_50_ value of 57.64 μg/ ml inhibition in MDA-MB-231 cells (Table 5). The increase in cell death with a rise in the dosage of test compounds signifies that methanolic leaf extract of the *C. roseus* plants was found active (Fig. 6a and b). The bioactive components of *C. roseus* belong predominantly to the seven phytochemical classes that are well-known to exhibit cytotoxicity. Possibly, the effectiveness of the CrPLE is influenced by the differences present in the metabolism of the active compounds. Nonlinear regression is a form of regression examination in which experimental facts are modeled by a purpose. This reveals a nonlinear amalgamation of the model constraints and relies upon at least one free factor (Fig. 6a and b).

**Table 5:** Cytotoxicity studies of the methanolic leaf extracts of *Catharanthus roseus* against the MDA-MB-231 Cell lines

Samples	Concentration μg/ ml	OD @ 590 nm	% Inhibition	IC_**50**_ μg/ ml
**Control**	0	0.743	0.00	**–**
**Vinblastine**	3.125	0.599	19.38	**21.54**
	6.250	0.560	24.63	
	12.500	0.437	41.18	
	25.000	0.292	60.70	
	50.000	0.165	77.79	
	100.000	0.092	87.58	
**Methanolic extract of** ***C. roseus***	10.00	0.614	17.36	**57.64**
	20.00	0.534	28.13	
	40.00	0.442	40.51	
	80.00	0.344	53.70	
	160.00	0.246	66.89	
	320.00	0.169	77.25

#### Mechanism of cell death: apoptotic assay

**Fig. 7:**
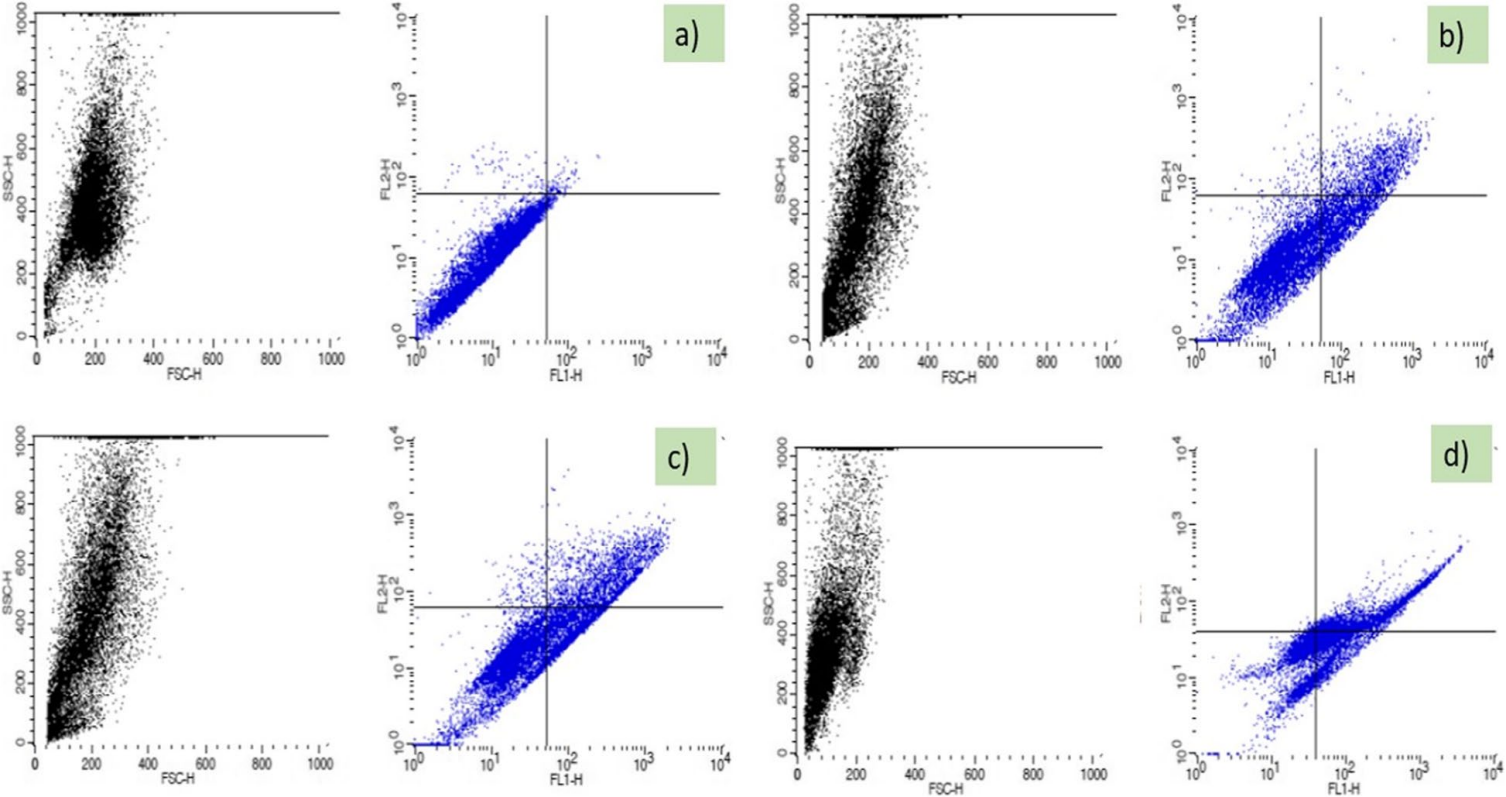
Graph of flow cytometry of MDA-MB-231 Cell lines with Annexin-V FITC where the cells were analyzed with **a** untreated (control); **b** treated with the *Catharanthus roseus* plant leaves extract (CrPLE) of 40 μg/ml; **c** 80 μg/ml; and **d** treated with standard Vinblastine (25 μM). In all the graphs, lower left (LL) indicates the living (viable) cells; lower right (LR) indicates early apoptotic cells; upper right (UR) indicates the late apoptotic cells and upper left (UL) indicates the necrotic cells

A diagram of the flow cytometry investigation of MDA-MB-231 cells with Annexin-V fluorescein isothiocyanate (FITC) of which, in the lower left corner are the living cells (Fig. 7). By understanding Fig. 7a, b, c and d, we can see distinct that all the cells experience a programmed cell death trail as designated through the occurrence of the small amounts of cells in each plot from the diagram. *Catharanthus roseus* plant leaves extract (CrPLE) treated at 40 μg/ml and 80 μg/ml has induced early and late programmed cell death in MDA-MB-231 cells with 20.59, 20.90% of early apoptotic cells and 13.17, 22.89% late apoptotic cells respectively (Fig. 7 and Table 6). Standard Vinblastine showed 35.75% early apoptotic and 25.58% late apoptotic cells in MDA-MB-231 cells. Thus, *C. roseus* plant leaves extract (CrPLE) can probably modify the programmed cell death protein expression and activate apoptosis in MDA-MB-231 cells.

**Table 6:** Flow cytometry analysis of apoptosis detection of MDA-MB-231 cells

Fluorescence activated cell-sorting analysis of apoptosis detection in MDA-MB-231 cells					
Samples	Samples (μg/ ml)	Viable cells	Early Apoptotic	Late Apoptotic	Necrotic cells
**Control**	–	97.56	00.89	00.84	0.71
**CrPLE (μg/ ml)**	40	65.23	20.59	13.17	1.01
**CrPLE (μg/ ml)**	80	54.56	20.90	22.89	1.65
**Standard Vinblastine (μM)**	25	38.16	35.75	25.58	0.51

## Discussion

The Indian subcontinent has the greatest richness and highest diversity in the flora of flowering medicinal plants. Most of the world’s population is mainly basically relying on therapeutic plants, the most extraordinary source is lifesaving drugs. About 80% of the world population takes an interest in the utilization of customary medication founded on plant materials, the therapeutic plants assume a powerful part in human medical care. They stay to be a significant useful help for working on the soundness of individuals and in the better treatment of several diseases.

### Characterization of the CrPLE

The phytochemicals present in methanolic leaf extracts of the *C. roseus* plants had exposed the existence of seven phytochemicals for example alkaloids, carbohydrates, flavonoids, glycosides, phenols, steroids as well as tannins indicating that the rich presence of auxiliary metabolites. This is in understanding that of prior investigations by different workers [21, 22, 40, 41, 63]. Comparable phytoconstituents present in the raw extracts were recorded in the *C. roseus* with different plant parts like leaves by Govindasamy and Srinivasan [17] and Wagay et al. [63], stem by Pham et al. [49], flower by Govindasamy and Srinivasan [17], root by Govindasamy and Srinivasan [17], and stem bark by Rose and Priya [55].

*Catharanthus roseus* is one of the significant therapeutic plants, very much perceived in Ayurveda and other customary treatment systems. This is recognized for its antitumor, antidiabetic, antimicrobial, antioxidant, and antimutagenic effects [40, 41]. As per Govindasamy and Srinivasan [17], the antibacterial, antitumor, anti-inflammatory, and antimicrobial activities of the plant concentrates are due to the presence of alkaloids. Accordingly, this plant portion of *C. roseus* displayed the presence of carbohydrates as well as phenols as a typical phytochemical.

*Catharanthus roseus* is notable for its pharmacological significance as it creates a wide spectrum (≥130) of terpenoid indole alkaloids (TIAs) whereas just *C. roseus* is known for creating such a wide scope of alkaloids and they are extremely interesting to be delivered by some other plants in such amounts as produced in *C. roseus*. These alkaloids contain vinblastine and vincristine generally utilized in anticancer chemotherapies [60].

The retention factor (Rf) value of the plant extracts ranged from 0.77 to 4.47 and colors fluctuated from brown to green. Yellow spots were recognized in the unrefined methanol extracts [63]. The therapeutic cost of monoterpene indole alkaloids (MIAs) such as 3′,4′-anhydrovinblastine, in addition to it as well as the substance intricacy has invigorated broad endeavors to comprehend the biochemical and molecular pathways engaged with their biosynthesis in the *C. roseus* plant source [25]. The methanolic extract of *C. roseus* contains the greatest elevated Rf value (0.70) and exhibited greyish color which demonstrates the existence of vindoline [22]. By looking at the specific bands and Rf values obtained by Kale et al. [22], we have determined the bands obtained from our present study. The methanolic extract of *C. roseus* comprises the Rf value of 0.77 which compares to the presence of greyish color demonstrating the occurrence of vindoline. The methanolic extract of *C. roseus* plant produces the typical light blue color under UV light (365 nm) and contrasted colors oscillated from brown to green observed under visible light. This is in agreement with the earlier discoveries by Nisar et al. [40, 41]. It is very utilized in biochemical investigation, for example, partition or seclusion of plant metabolites from the methanolic extracts of *C. roseus* plants.

The absorption maxima in UV-spectrophotometric investigation were recorded at 250 nm aided in simple examination employing HPLC analysis. HPLC examination showed that several noticeable mixtures contained in the unrefined extract were available in the methanolic fraction, in which catechin and caffeic acid were recognized as significant. i.e., the CrPLE of catechin and caffeic acid were present in the low concentration when contrasted with the principles. In contrast to the findings of Goboza et al. [16], the CrPLE of caffeic acid was 0.614 which is a low concentration when contrasted with our outcomes (1.807). This showed that we have obtained the results screening the threefold concentration of caffeic acid in the CrPLE to that of Goboza et al. [16].

HPLC chromatogram of the methanolic extract disclosed the higher dosages of the selected phenolic compounds. Polyphenolic compounds are available in plant sources as optional metabolites as well as their incorporation in human food has been documented to have amazing medical advantages. Polyphenolic compounds can search for reactive oxygen species (ROS) and reactive nitrogen species (RNS) in natural frameworks. A measurable polyphenolic evaluation of the different concentrates of *C. roseus* uncovered that methanolic extract was the top dissolvable to remove the phenolic compounds. These discoveries forms concurrence as per past examinations by Rasool et al. [54] and Kabesh et al. [21] that reported high polyphenolic content and antioxidant action are available in methanolic extract of *C. roseus*. Thus, the utilization of therapeutic plant sources in the treatment of several diseases is credited with the existence of chemical ingredients such as phenolic substances and alkaloids in *C. roseus* that have various attributes which add to the helpful impacts of plants. This proposes that the biomolecules acquired from the CrPLE may perhaps act as oxidizing/ reducing agents/ specialists utilized for different applications. Hence, the current study was valuable and supportive for the uses of medications, plans, restorative, and possible proposals for the assembling of therapeutic agents and agricultural products.

### Medicinal practices of the biologically synthesized CrPLE

Drug plants assume a critical part in the revelation of new medications utilized in flow medication and tracked down a powerful medication for different diseases. Antimicrobial susceptibility studies were directed against gram-negative (−) bacteria such as *Escherichia coli*, gram-positive (+) bacteria for example *Staphylococcus aureus*, and fungi for instance *Candida albicans* and *Aspergillus niger*. The methanolic leaf extract of the *C. roseus* (CrPLE) revealed strong inhibitory activity against the microorganisms such as the gram-negative (−) bacteria – *E. coli*, the gram-positive (+) bacteria – *S. aureus*, and fungi – *C. albicans* and *A. niger*. Tested CrPLE disclosed intense antimicrobial action compared to all organisms. The zones of inhibition showed moderate to highest inhibitory activity against the methanolic leaf extracts from the *C. roseus* plants and exhibited more than 50% inhibitory action towards these mentioned organismal strains. Consequently, the methanolic leaf extract of *C. roseus* plants unveiled the highest antimicrobial activity and established fatal outcome for *S. aureus* at various doses. Consequently, our study has shown that the CrPLE displayed the most noteworthy antimicrobial activities such as antibacterial and antifungal actions, and hence, could be helpful in natural applications. Accordingly, alcoholic extract of *C. roseus* could be utilized as a likely antimicrobial source [15, 17, 22, 48, 49, 63]. Similar kinds of results were obtained by Khalil [24] from the investigation of the antimicrobial action from ethanol leaf extract of *C. roseus* towards some human pathogenic microorganisms (*S. aureus* and *E. coli*) as well as pathogenic fungi (*C. albicans*) with slight variation. The sturdiest inhibition movement of the leaf extract was detected towards the *S. aureus* (15 mm zone) at 100 mg/ml of leaf extricate antimicrobial action of this plant.

*Catharanthus roseus* is an important therapeutic plant source for drugs since the majority of bacterial microbes are creating opposition against large numbers of presently accessible antimicrobial medications. Plants have ended up being critical normal assets for compelling chemotherapeutic specialists and offering a wide range of action with more prominent accentuation on the preventive activity. The anti-cancer and antimicrobial properties of *C. roseus* have been examined the microorganisms such as *Pseudomonas aeruginosa* NCIM 2036, *Salmonella typhimurium* NCIM 2501, and *Staphylococcus aureus* NCIM 5021. So, extracts from these plant leaves can be utilized as a prophylactic specialist for a large number of pestilence infections [45, 48, 49, 63].

Because of the attribution of optional metabolites of different synthetic compounds existing in the *C. roseus* plant material either separately, the antimicrobial action found in this current study. The discovery of a strong cure from the plant beginning will be an incredible movement in microbial contamination treatments. This study offers help to the plant’s customary and elective use against different diseases and infections. Additionally, the utilization of regular items has been urged because of less or no aftereffects, cost adequacy, and improvement of protection from traditional engineered antibiotics. Henceforth, this study holds significance in involving therapeutic plants as an elective hotspot for treating different diseases [21]. Supplementary examinations are expected to detach the bioactive constituents answerable for the noticed antimicrobial movement. Nonetheless, extra examinations utilizing the carcinoma cell line are important to know the impacts of CrPLE answerable for the cytotoxic action.

Cytotoxicity assay has turned into a significant examination for showing a compound of pharmacological significance. The methanolic extracts produced from *C. roseus* plant leaves presented a cytotoxic interaction against MDA-MB-231 cell lines. The samples of CrPLE for 24 h treatment showed an IC_50_ value of 57.64 μg/ml inhibition in MDA-MB-231 cells. The expansion of cell demise with an expansion in the grouping of test intensifies connotes that the methanolic extracts of the *C. roseus* plants were seen as successful. Further, the differences in the IC_50_s of the CrPLE *in vitro* were reflected in the effectiveness of the extracts in the MDA-MB-231 cell line model. Bioactive components of the genus *Catharanthus* have a place overwhelmingly with the seven phytochemical classes that are not able to display cytotoxicity [49]. Perhaps, the viability of the plant concentrates might be affected by the auxiliary metabolites present in the digestion cycle of the dynamic mixtures.

It exhibited that the n-butanol fraction confined the most elevated degrees of saponins and phenolics, and had the most grounded cancer prevention agent limit among the tried CrPLE [49]. The n-butanol fraction additionally required solid cytotoxic exercises *in vitro* on a wider scope of malignant growth cell lines including A2780 (ovarian), H460 (lung), A431 (skin), MIA PaCa-2 (pancreas), Du145 (prostate), HT29 (colon), MCF-7 (breast), BE2-C (neuroblastoma), SJ-G2, U87 and SMA (glioblastoma) at small amounts (GI50 values of 5.2–21.0 μg/mL) [49]. Like the consequences of n-butanol portion techniques, our methanolic acquired obtained from the CrPLE is likewise a rich wellspring of bioactive mixtures that can be confined for their possible use as antimicrobial medications or antitumor remedial specialists. *Catharanthus roseus* is also likewise has a perceived as “magic plant”, “flower of immortality” by the Germans, “the flower of death” by the Italians, the “violet of the sorcerers” by the French, and “an emblem of friendship”. Henceforth, the plant parts (aerial and underground) removed are being utilized compared to a few significant disorders [49, 56].

From apoptotic examination by flow cytometry, CrPLE might conceivably change the apoptotic protein articulation and activate apoptosis in MDA-MB-231 cells. Comparable sort results were acquired from examinations directed by Widowati et al. [66]. *Catharanthus roseus* comprises a scope of dimeric indole alkaloids with huge antitumor exercises. These alkaloids have been originated to have programmed cell death-actuating action towards tumor cells *in vitro* and *in vivo* facilitated by nuclear factor kappa-light-chain-enhancer of activated B cells (NF-κB) and c-Jun N-terminal kinase (JNK) pathways, in which DNA injury and mitochondrial brokenness assume significant parts. In this manner, the acceptance of programmed cell death through cathachunine occurred through a ROS-dependent mitochondria-mediated intrinsic pathway as opposed to an outward pathway and was managed by the Bcl-2 protein family. It applied a powerful antitumor impact on human leukemia cells by the enlistment of programmed cell death and employing an inborn trial [64, 65].

Therapeutic plants are utilized in the conventional treatment of individuals in India for quite a long while because of homegrown items with fewer aftereffects and are financially savvy. *Catharanthus roseus* plants comprise different alkaloids, carbohydrates, saponin, flavonoids and phenolics, and phytochemicals such as vincristine, raubasin, vinblastine, vincolinine, vinacardine, leurocristine, catharanthamine, etc.. It had revealed the existence of different pharmacological activities for instance anticancer, antidiabetic, antiulcer, antioxidant, antimicrobial, antihyperglycemic, antihypertensive, antidiabetic, and wound healing, etc. [43]. *Catharanthus roseus* is a significant evergreen therapeutic spice utilized for the most part for therapy of malignant growth of cancer and diabetics. Researchers have gathered data on the conventional purposes, phytochemical ingredients, and pharmacological possessions of plants. Along these lines, they have perceived the well-being-advancing activities of this versatile plant and it could likewise give hints to the disclosure of a novel principal substance of drug significance [29].

The inferences of the study affirmed the remedial strength of *C. roseus* plant’s roots, stems, leaves, and flowers can be utilized in conventional medication and progressed pharmacology, and gives the promising lead to the revelation of intense antimicrobial mixtures in helpful and dietary use all around the world. The outcome of the current examinations comprises strong proof to approve folkloric utilization of these *C. roseus* plants as a solution for different diseases. Consequently, the current study has been valuable and accommodating for the utilization of drugs, therapeutic and expected applications, and in the assembling of different herbicides, pesticides, and agricultural fields.

## Conclusions

Description of methanolic leaf extracts of *C. roseus* plants manages documentation for various auxiliary metabolites, wherein they assume a noticeable part as industrially significant naturally dynamic particles having different pharmacological possessions. *Catharanthus roseus* also exhibited ordinary anti-microbial activity, and cytotoxic and anticancer properties. Besides, this plant species showed a critical cytotoxic impact of methanolic leaf extracts on the MDA-MB-231 cell lines. This could be additionally affirmed that phytochemical constituents found in the *C. roseus* plant may perhaps add to the current cytotoxicity. The present study gave important data on phytochemistry, hostile to microbial action and cytotoxicity of *C. roseus*, and the necessity for the extra need for additional investigation of DNA discontinuity and caspase measure studies, pharmacology, and toxicology, in a trial creature model.

## Data Availability

The datasets used and/or analysed during the current study are available from the corresponding author on reasonable request.
